# Insecticidal effect of ethnobotanical plant extracts against *Anopheles arabiensis* under laboratory conditions

**DOI:** 10.1186/s12936-021-04004-6

**Published:** 2021-12-14

**Authors:** Desta Ejeta, Ansha Asme, Animut Asefa

**Affiliations:** Department of Biology, College of Natural and Computational Science, Assosa University, Assosa, Ethiopia

**Keywords:** *Anopheles arabiensis*, Botanical insecticide, Dangur, Ethnobotanicals, Malaria

## Abstract

**Background:**

The emergence and spread of resistant strains of malaria vectors to chemical insecticides are becoming major problems for malaria vector management. Natural plant products have a vital role to play in the current challenge of malaria control. The current study was conducted to evaluate insecticidal effect of ethnobotanical plant extracts against the primary malaria vector, *Anopheles arabiensis* in northwestern Ethiopia.

**Methods:**

Primarily, ethnobotanical plants used for *Anopheles* mosquito control were surveyed in Dangur district, northwestern Ethiopia. Insecticide-susceptible strains of *Anopheles arabiensis* mosquito were reared in the insectary of the Tropical and Infectious Diseases Research Centre, Assosa University. After surveying plants used for mosquito control in local people, four frequently used plants were identified for extraction. The larvicidal and adulticidal potential of frequently used plant extracts against susceptible strains of the laboratory colony were evaluated.

**Results:**

A total of 15 plants were identified as ethnobotanical plants that help local people with mosquito control. *Azadirachta indica, Ocimum lamiifolium, Ocimum americanum, Moringa olifeira* leaf, and *Moringa olifeira* seed species of local plants were found to be frequently used to kill and/or repel mosquitoes in the study district. All the plant extracts were found to have potential larvicidal activity against fourth instar larvae of *An. arabiensis* and only ethanol and methanol extract of *Azadirachta indica* and *Ocimum lamiifolium* were found to have potential adulticidal effect against adult of *An. arabiensis*. The highest larvicidal activity was observed in ethanol extract of *Azadirachta indica* with 95% larval mortality and lowest Lethal Concentration 50 (LC 50) of 40.73parts per million (ppm) and LC90 of 186.66 ppm. The highest adulticidal activity was observed in methanol extract of *Azadirachta indica* with 75% adult mortality at 300 ppm and lowest LC50 of 106.65 ppm and LC90 of 1,293 ppm. The lowest larvicidal and adulticidal activity was observed in methanol extracts of *Ocimum lamiifolium* with 63.35% larval mortality and leaf extract of *Moringa olifeira* with 50% adult mortality at 300 ppm, respectively.

**Conclusion:**

Ethanol extract of *Azadirachta indica* exerted a remarkable larvicidal effect against *An. arabiensis* and thus it can be used for botanical mosquito insecticide development. Since the current finding is based on susceptible strain of *An. arabiensis*, further work on wild mosquitoes is recommended.

## Background

*Anopheles arabiensis* is the principal malaria vector in Ethiopia, having a wide distribution while *Anopheles pharoensis, Anopheles funestus*, *Anopheles nili*, and *Anopheles stephensi* have a secondary role in malaria transmission in the country [1–3]. Vector control is a crucial prevention tool to mitigate the disease. Long-lasting insecticidal nets (LLINs) and indoor residual spray (IRS) are among the most effective malaria vector management strategies recommended in Africa. However, the wide spread of insecticide-resistant strains of *Anopheles* is challenging chemical insecticide-based malaria control strategies. Insecticide susceptibility tests carried out in Ethiopia have shown different levels of resistance by the principal vector to insecticides in use for IRS and/or to treat LLINs [4, 5]. For these reasons, looking for an alternative is becoming of major interest to scientists and policymakers working in the area.

Traditionally or culturally, different communities use different plants in various forms to protect themselves against mosquitoes and other insect bites [6, 7]. Naturally occurring compounds and their derivatives are of increasing interest for the development of new insecticidal compounds against malaria vectors. Plants possess a wide range of bioactive phytochemicals that are selective, biodegradable and have minor or no adverse effects on non-target organisms and the environment [8]. Reports indicate that essential oils and extract of local plants have a promising larvicidal, adulticidal and repellent activity against malaria vectors [9, 10]. Gathering information about ethnobotanical plants used in a particular society and evaluating efficacy is important. The main objective of the present study was to investigate the insecticidal effect of ethnobotanical plant extracts against *An. arabiensis* under laboratory conditions.

## Methods

### Study area

**Fig. 1:**
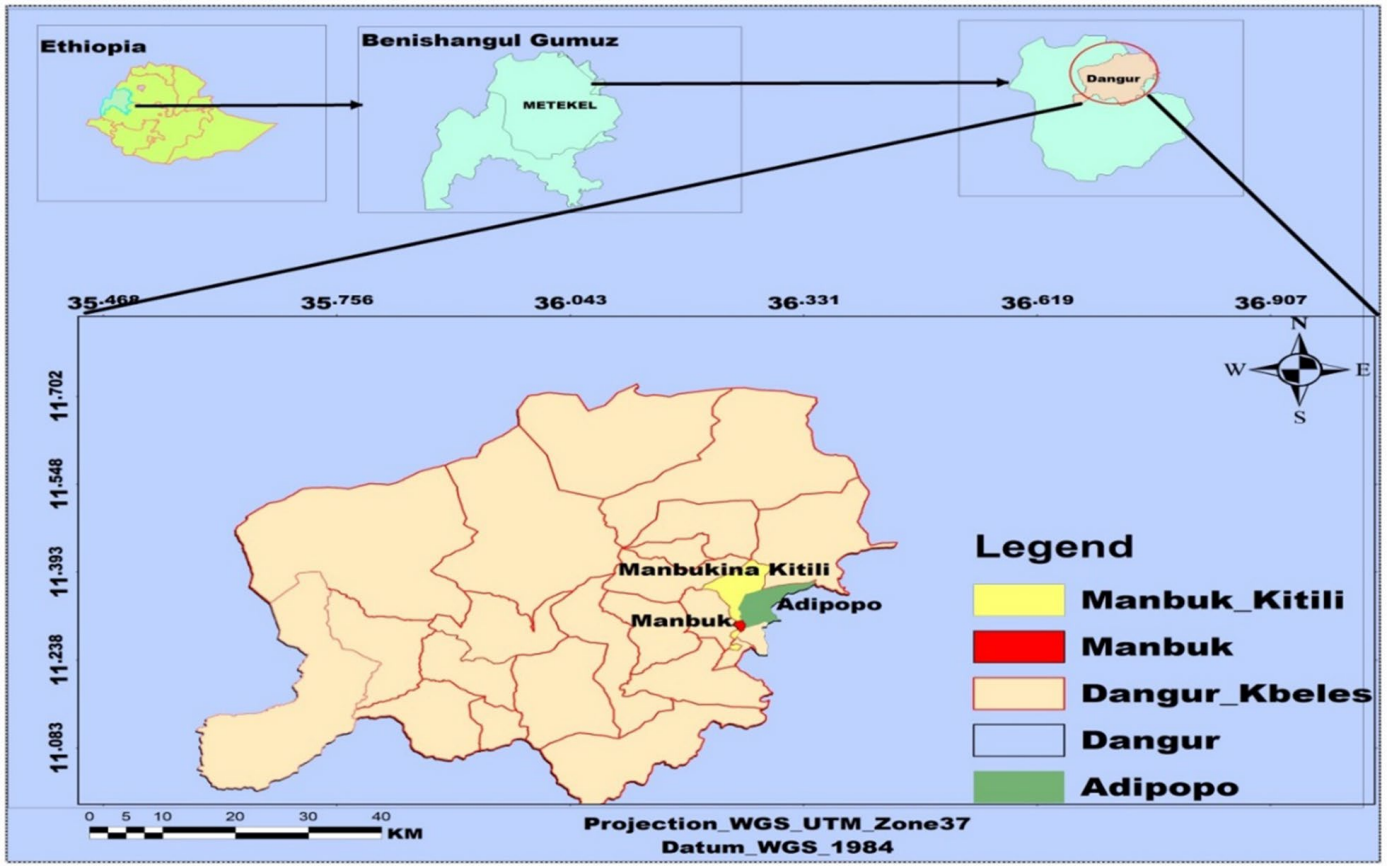
Map of the study area

Ethnobotanical collection was conducted in three purposively selected villages: Manbuk 01, Kitil and Adipopo, Dangur district, northwestern Ethiopia. Dandur district is situated 687 km from the capital city, Addis Ababa, in northwest Ethiopia (Fig. 1). The district has an estimated total population of 68,653. Geographically, the district lies at latitude and longitude of 11°30′N and 35°50′E, respectively, with elevation ranging from 672 to 2731 m above sea level. Based on the information from metrological data in 2021, the area has mean annual rainfall ranging from 700 to 1400 mm per year and mean annual temperature ranging from 26–35 °C per year.

### Ethnobotanical data collection techniques

Semi-structured interviews were prepared for inhabitants/key informants of the three villages (Manbuk 01, Kitil, Adipopo) and were used as a guide following Martin [11]. A total of 20 key informants were identified based on the recommendations of local authorities and knowledgeable elders who were suggested by respective village elders. Key informants were interviewed to identify plants they use to repel and/or kill insects in general and mosquitoes in particular.

### Voucher specimen collection and identification

Voucher specimens of the reported plants as repellant against mosquito vectors were collected and identified with taxonomic keys [12].

### Preparation of crude plant extracts

Plant extraction was conducted in the microbiology laboratory, Assosa University. The leaves and seeds of the plants were washed thoroughly using clean water and air-dried under shade at room temperature for 15 days. The dried materials were ground separately to powder using a grinding mill. The powdered material was macerated with ethanol and methanol using Erlenmeyer flasks and placed on an orbital shaker at 60 rpm at room temperature for 72 h [13]. The plant extract was filtered through cotton and subsequently with Whatman filter paper (12.5 cm size). Filtrates were concentrated using a rotary evaporator to remove solvents from the extract. The crude extracts were collected in small volume beakers and kept in the freezer until used for mosquito efficiency tests.

### Mosquito rearing

Larvae of *An. arabiensis* were obtained from the Tropical and Infectious Diseases Research Centre’s insectary laboratory, Assosa University. Mosquitoes were reared using standard procedures and maintained at 25 ± 2 °C temperature and 80 ± 10% relative humidity and 12:12 light and dark photoperiod. Larvae were fed with yeast a 3:1. When pupae were formed, no food was supplied, and they were transferred to a cup that contained water by disposable pipettes and put in screened cages for adult emergence. The adults were fed on 10% glucose solution soaked in cotton pads, in addition to an animal (rabbit with shaved back and belly) blood meal given to the females twice per week. A petri dish lined with a moist cotton piece and covered with filter paper was put inside each cage for egg laying [14], which ensured that fourth instar larvae and 2–5 days old adult mosquitoes were continuously available for different bioassay tests.

### Larvicidal activity

#### Preparation of test and control solutions

Two-hundred mg of the dried crude ethanol and methanol extract of each plant were placed in a standard measuring flask with distilled water to prepare 20 ml of 1% stock solution; 0.1 ml of Tween 80 was used as an emulsifier. From the 1% stock solution, concentrations of 50, 100, 150, 200, 250, and 300 ppm were prepared by adding the appropriate volume of dilution; 0.1 ml of Tween 80 was made up to 100 ml distilled water to serve as the negative control solution [14].

#### Larvae bio-assay with crude leaf extract

In the first phase of bio-assay, mosquito activity to frequently used plants, ethanol and methanol of crude extracts of the leaves and seed, were screened at 300 ppm concentration. Batches of 20 fourth instar larvae of *An. arabiensis* were transferred using a dropper to 200 ml glass beakers each containing 100 ml water and one batch as a negative control for each concentration. Each test was run three times. The test containers were held at 25 ± 2 °C and 80 ± 10% relative humidity with a photoperiod of 12 h light followed by 12 h dark. Larvae mortality was recorded after 24 h exposure in each concentration of test solutions [14].

#### Determination of LC50 and LC90 of the crude leaf and seed extract of test plants

Based on the preliminary screening, ethanol and methanol of the leaf and seed of plant extract was selected and subjected to dose–response bio-assay at concentrations of 50, 100, 150, 200, 250 and 300 ppm. The average mortality after 24 h was recorded and used to determine LC50 and LC90 values [14].

### Adulticidal activity

#### Adult bio-assay with crude leaf extract

The triplicate series contained 20 females of *An. arabiensis* in each tube. In the first phase of bio-assay, the mosquito activity of ethanol and methanol crude plant extracts was screened at 300 ppm concentration**.** This concentration was impregnated into filter papers (12 × 15 cm). Distilled water only was used as a negative control. The impregnated papers were air-dried for 5 min and then inserted into an exposure tube in the WHO testing kit. Twenty 2–5 days old, blood-starved, female mosquitoes were introduced into the holding tube and held for 1 h to acclimatize. The mosquitoes were transferred by gentle blowing in the exposure tube. After 1 h in the exposure tube, mosquitoes were transferred back to the holding tube to recover. A pad of cotton soaked with 10% glucose solution was placed on the mesh screen to feed recovering experimental and control mosquitoes. At the end of the 24 h recovery period, mosquito mortality was recorded and the percentage mortality was calculated [15].

Percentage of mosquito mortality was calculated by using the formula:

$${\text{Percentage of mortality}}=\frac{{{\text{Number of dead mosquitoes}}}}{{{\text{number of mosquitoes tested}}}}*100$$

#### Determination of lethal concentration 50 and lethal concentration 90 of the crude leaf extracts of test plants

Based on the preliminary screening, ethanol and methanol leaf of plant extract was selected and subjected to dose–response bio-assays at concentrations of 50, 100, 150, 200, 250 and 300 ppm. The average mortality after 24 h was recorded and used to determine LC50 and LC90 values [14].

#### Phytochemical screening of methanol and ethanol of crude extracts of test plants

Crude methanol and ethanol extracts of all test plants were subjected to test the presence of major secondary metabolites (alkaloids, flavonoids, terpenoids, tannin, saponin, phenols following the procedures described by Madike et al. [16] and Bandiola et al. [17].

### Data analysis

Data from all replications were pooled and mean per cent mortalities of larva and adult mosquitoes treated with crude leaf extract of the plants were determined by analysis of variance (ANOVA) using SPSS version 20. Fishers Least Significant Difference (LSD) was used to investigate statistical significance between the different test plants against mosquito mortality. The difference between means was considered statistically significant at *P* < 0.05. The LC50% and LC90% and other statistics at 95% fiducial limit of lower confidence limit and upper confidence limit and Chi-square values were determined using dosage mortality probit regression analysis of SPSS program version 20 to determine their larvicidal and adulticidal efficacies [14].

## Results

### Ethnobotanical plant species used to repel mosquitoes in the study area

A total of 15 plant species used to prevent mosquito bites were collected and identified from the study area (Table 1). From all plant species collected and identified, the most frequently used plants (*Azadirachta indica* (75%), *Ocimum lamiifolium* (65%), *Ocimum americanum* (70%), leaf of *Moringa olifeira* (50%), and seeds of *Moringa olifeira* (50%)) were selected to test their larvicidal and adulticidal effects against *An. arabiensis.*

**Table 1:** Plant species collected from the three villages of Dangur district

Scientific name	Vernacular name	Family name	Plant type	Part used and methods of application	Usage frequency (%)
*Allium sativum* L.	Nech Shenkurt (A)	Alliaceae	Herbs	Crushing 3–5 bulbs and applying juice to the body	5
*Azadirachta indica* A.	Meem(A)Lemima(G)	Meliaceae	Tree	10–15 leaves put in the house	90
*Carica papaya* L.	Papayaa(A)	Caricacae	Tree	5 leaves crushed and apply juice to exposed parts of the body	10
*Citrus sinensis* L.	Birtukan(A)	Rutaceae	Tree	Fruit peeled and burned to generate smoke	15
*Crotonmacrostachyus *Hochst. Ex Del.	Bissana(A)	Euphorbiaceae	Tree	Burn 8–10 dried leaves to generate smoke	5
*Cymbopogon citratus* Stapf.	Tej sar(A)	Poaceae	Herb	Burn 10 of leaf plant to generate smoke	10
*Echinops keberich* Mesfn.	Kebericho(A) Asitana(G)	Asteraceae	Herb	3 dried root parts burned to generate smoke	25
*Eucalyptus globulus* L.	Nech bahirzaf(A)	Myrtaceae	Tree	Burn whole plant to generate smoke	10
*Eucalyptus camaldulensis* Dehn.	Key beharzaf(A)	Myrtaceae	Tree	Burn whole plant to generate smoke	10
*Moringa olifeira* L*.* leaf	Sheferaw(A) Chehwie(G)	Moringaceaea	Tree	15–20 leaves put in the house	50
*Moringa olifeira* L*.* seed	Sheferaw(A) Chehwie(G)	Moringaceaea	Tree	20–30 seeds crushed and rubbed on skin	50
*Ocimum lamiifolium* Hochst, ex Benth.	Damakasse(A)Akawaya(G)	Lamiaceae	Shrub	About 5 leaves—juice applied to the skin	65
*Ocimum americanum* L.	Yezenjero Zekakebie(A)Omasiya(G)	Lamiaceae	Shrub	About 5 leaves—juice applied to the skin	70
*Olea europaea *L.	Woira(A)	Oleaceae	Tree	3 stem parts of plant burned to generate smoke	20
*Otostegia integrifolia *Benth.	Tnjut(A)	Lamiaceae	Shrub	Dry 10 leaves and smoke	25
*Ruta chalepensis* L.	Tena Adam (A)	Rutaceae	Herb	Thermal expulsion and direct burning of seeds	20

*A* Amharic, *G* Gumzegna

### Larvicidal activity of crude leaf and seed extracts of test plants

The highest mortality was recorded in ethanol leaf extract of *Azadirachta indica* (95%). Methanol leaf extract of *Ocimum lamiifolium* recorded the lowest activity with mortality of 63.35% (Table 2). There was no statistically significant (p > 0.05) difference in the larvicidal potential between ethanol and methanol extract of *Moringa olifeira* leaf and *Moringa olifeira* seed. However, there was a significant difference (p < 0.05) in the larvicidal efficacy between ethanol and methanol leaf extracts of *Azadirachta indica, Ocimum lamiifolium,* and *Ocimum americanum.*

**Table 2:** Larvicidal activity of ethanol and methanol crude leaf and seed plant extract of test plants against fourth instar of larvae of *Anopheles arabiensis* at 300 ppm

% Mean mortality ± SE		
Plant species	Solvent	
	Ethanol	Methanol
*Azadirachta indica*	95 ± 0.577^Aa^	65 ± 1.528^Ba^
*Ocimum lamiifolium*	90 ± 0.577^Aab^	63.35 ± 3.480^Bab^
*Ocimum americanum*	88.25 ± 0.333^Aabc^	76.65 ± 0.667^Babc^
*Moringa olifeira* leaf	86.65 ± 0.667^Abc^	91.65 ± 0.882^Ac^
*Moringa olifeira* seed	83.35 ± 0.333^Abc^	90 ± 0.557^Ac^
Negative Control	0.00 ± 0.000^Ad^	0.00 ± 0.000^Ad^

*Each value (% mean ± SE) represents mean value of three replicates

*Means followed by the same letters within the same row (Upper case) and within the same column (Lower case) are not significantly different (p > 0.05)

### Determination of LC50 and LC90 of leaf and seed crude plant extract

Ethanol leaf extract of *Azadirachta indica, Ocimum lamiifolium, Ocimum americanum,* and methanol extract of *Moringa olifeira* leaf and *Moringa olifeira* seed were subjected (Table 3). Ethanol leaf extract of *Azadirachta indica* caused the highest larvicidal activities against the fourth instar larvae of *An. arabiensis* with the lowest LC50 of 40.73 ppm and LC90 of 186.66 ppm. Ethanol crude leaf extract of *Ocimum americanum* showed the relatively highest LC50 and LC90 (LC50 = 92.42 ppm, LC90 = 551 ppm).

**Table 3:** LC50 and LC90 of test plant extract against larvae of *Anopheles arabiensis*

Solvent	Plant name	LC 50	LCL	UCL	LC90	LCL	UCL	X^2^ (df^b^ = 4)
Ethanol	*Azadirachta indica*	40.73	10.38	65.31	186.66	131.97	378.50	0.339
	*Ocimum lamiifolium*	40.93	5.35	69.23	230	170.5	944.18	0.744
	*Ocimum americanum*	92.42	45	129.22	551	318	3092	1.419
Methanol	*Moringa olifeira* leaf	45.43	11.93	72	260	159	558	0.390
	*Moringa olifeira* seed	62.35	19.1	94.01	388	239.75	1792.11	2.831

^*^LC50-Lethal concentration kills 50% of the exposed larvae

LC90-Lethal concentration kills 90% of the exposed larvae

*UCL* Upper confidence limit

*LCL* Lower confidence limit

*X*^*2*^ Chi-square

*df* degree of freedom

### Adulticidal activity of crude leaf and seed extracts of test plants

The highest mortality was recorded in methanol leaf extract of *Azadirachta indica (*75%) (Table 4). However, the ethanol and methanol leaf extract of *Moringa olifeira* was found to have lowest mortality at 50% (Table 4). There was no statistically significant (p > 0.05) difference in the adulticidal potential between ethanol and methanol extract of all test plants. However, there was a significant difference (p < 0.05) in the adulticidal efficacy between ethanol leaf extracts of *Azadirachta indica* and *Ocimum americanum* and between ethanol leaf extracts of *A. indica* and *M. olifeira* leaf and between ethanol leaf extracts of *A. indica* and seed extract of *M. olifeira.*

**Table 4:** Adulticidal activity of ethanol and methanol crude plant extract of test plants against adult of *Anopheles arabiensis* at 300 ppm

% Mean mortality ± SE		
Plant species	Solvent	
	Ethanol	Methanol
*Azadirachta indica*	71.65 ± 0.333^Aa^	75 ± 0.577^Aa^
*Ocimum lamiifolium*	70 ± 0.577^Aa^	65 ± 1.201^Aa^
*Ocimum americanum*	55 ± 0.577^Ab^	51.5 ± 0.333^Ab^
*Moringa olifeira* leaf	50 ± 0.577^Ab^	50 ± 0.577^Ab^
*Moringa olifeira* seed	55 ± 0.881^Ab^	53.3 ± 1.4529^Ab^
Negative Control	0.00 ± 0.000^Ac^	0.00 ± 0.000^Ac^

^*^Each value (% mean ± SE) represents mean value of three replicates

^*^Means followed by the same letters within the same row (Upper case) and within the same column (Lower case) are not significantly different (p > 0.05)

### Determination of LC50 and LC90 of crude leaf plant extracts

Methanol leaf extract of *Azadirachta indica* caused the lowest LC50 of 106.655 ppm and LC90 of 1,293 ppm and ethanol crude leaf extract of *Azadirachta indica* showed the highest LC50 and LC90 (LC50 = 151.033 ppm, LC90 = 1059 ppm) (Table 5).

**Table 5:** LC50 and LC90 of test plant leaf extracts against adult *Anopheles arabiensis*

Solvent	Plant name	LC50	LCL	UCL	LC90	LCL	UCL	X^2^(df^b^ = 4)
Ethanol	*Azadirachta indica*	151.033	96.794	232.69	1059	486.47	20,498.55	0.360
	*Ocimum lamiifolium*	125.48	50.472	206.825	1468.15	526.74	88,685.0	0.471
Methanol	*Azadirachta indica*	106.655	30.232	168.537	1293	481.79	751,031	1.099
	*Ocimum lamiifolium*	158.50	105.391	245.687	1054.1	491.37	17,355.7	0.045

*LC50-Lethal concentration kills 50% of the exposed adult

LC90-Lethal concentration kills 90% of the exposed adult

*UCL* Upper confidence limit

*LCL* Lower confidence limit

*X*^*2*^ Chi-square

*df* degree of freedom

### Phytochemical screening of methanol and ethanol crude leaf and seed extracts of test plants

Phytochemical screening was conducted for all methanol and ethanol crude plant extracts to test the presence of alkaloids, flavonoids, terpenoids, tannin, saponin, and phenols; the results are presented on Table 6.

**Table 6:** Phytochemical screening of methanol and ethanol crude extracts of test plants

Plant name	Solvent	Secondary metabolite					
		Alkaloids	Flavonoids	Terpinoids	Tannin	Saponin	Phenols
*Azadirachta indica*	Ethanol	+	+	+	+	+	+
	Methanol	+	+	+	+	+	+
*Ocimum lamiifolium*	Ethanol	+	−	+	+	+	+
	Methanol	+	−	+	+	+	+
*Ocimum americanum*	Ethanol	−	−	+	−	−	+
	Methanol	−	−	+	−	−	+
*Moringa olifeira* leaf	Ethanol	+	+	+	+	+	_
	Methanol	+	+	+	+	+	+
*Moringa olifeira* seed	Ethanol	+	+	+	−	−	−
	Methanol	+	+	+	−	−	+

* + * Present, *−* absent

## Discussion

A total of 15 local plant species were identified used for mosquito control in Dangur district. The presence of several plant species used for mosquito control by local people is a good indication of the deep-rooted culture of local plants in the study area. This result is similar to other studies of two plants conducted in Ethiopia [18, 19]. The current results show that local communities had indigenous knowledge and give emphasis to using local plants to repel mosquitoes. Out of all local plant species used for mosquito control the most frequently used were *Azadirachta indica* (75%), *Ocimium lamiifolium* (65%), *Ocimium americanum* (70%), leaf of *Moringa olifeira* (50%), and seeds of *Moringa olifeira* (50%). Some of the repellant plants recorded in the current study have been reported in other parts of Ethiopia [9, 20, 21].

All the test plants were found to have potential larvicidal activities against the fourth instar larvae of *An. arabiensis* at the test concentration. The highest mortality was recorded in ethanol leaf extract of *Azadirachta indica*, the methanol leaf extract of *Moringa olifeira,* the ethanol leaf extract of *Ocimum lamiifolium,* and methanol seed extract *Moringa olifeira.* However, the methanol leaf extract of *Ocimum lamiifolium, Azadirachta indica*, *Ocimum americanum* followed by ethanol seed extract of *Moringa olifeira,* the leaf extract of *Moringa olifeira,* and leaf extract of *Ocimum americanum* recorded the lowest insecticidal potential. The toxicity difference and extraction solvent among test plants suggested that different plants have different phytochemicals, which can be extracted by different solvents.

High larval mortality of 95% was caused by ethanol leaf extract of *Azadirachta indica*. This finding is similar to the report of Okumu et al. [22] where hexane leaf extract of *Azadirachta indica* was found to cause 100% mortality against larvae of *Anopheles gambiae* at 1,000 ppm although the concentrations are different. This could be due to the presence of excess bioactive secondary metabolites [23].

However, this finding disagrees with findings of Vilayatakar et al. [24] that reported the aqueous extract of *Moringa oleifera* leaves against larvae of *Anopheles* had shown 60% mortality at 500 ppm. Similarly*,* acetone extract of *Ocimum lamiifolium* oil was found to have high larvicidal activity against *An. arabiensis* [25]. Various factors might be responsible for the larvicidal activity, but the difference in larvicidal activities in the current finding could be due to locality of the plants, and different solvents.

Methanol leaf extract of *Azadirachta indica* causes adult mortality 75%, which could be due to the presence of bioactive secondary metabolites. This finding is in line with the earlier finding of Kamaraj et al*.* [26], who reported adulticidal efficacy of methanol against *Culex gelidus Theobald*.

Ethanol leaf extract of *Ocimum lamiifolium* and *Ocimum americanum* was found to have 71.65 and 55% mortality, respectively, at 300 ppm against adults of *An. arabiensis* after 24 h exposure. This result is lower compared to the finding of Messebo et al. [27], where ethanol leaf extract of *Ocimum lamiifolium* showed 90% mortality against adult *An. arabiensis* after 1 h exposure 0.25(v/v) in other parts of Ethiopia. The difference in adulticidal activity in the current finding could be due to species of plants, extraction solvent, and locality of the plants.

## Conclusions

Ethnobotanical plants are widely used in Dangur district, northwestern Ethiopia. *Azadirachta indica, Ocimum lamiifolium, Ocimum americanum, Moringa olifeira* leaf, and *Moringa olifeira* are plant species frequently used for mosquito prevention in northwest Ethiopia. They have effective larvicidal potential against fourth instar larvae of *An. arabiensis.* Ethanol leaf extract of *Azadirachta indica* has higher larvicidal potential and is promising for further botanical insecticide development. Ethanol and methanol extract of *Azadirachta indica* and *Ocimum lamiifolium* have potential adulticidal activity with > 60% mortality against adult *An. arabiensis*. The phytochemical analysis of leaf extract of *Azadirachta indica* has secondary metabolites, such as alkaloid, flavonoid, terpenoid, tannin, saponin, and phenols, which have potential insecticidal activities. Further study is recommended to identify the active ingredient of ethanol and methanol extracts of *Azadirachta indica* and their mode of action in the study area and other parts of Ethiopia.

## Data Availability

The datasets used and/or analysed during the current study are available from the corresponding author on reasonable request.
